# Population bottlenecks and temporal constraints shape dengue virus infection of *Aedes aegypti* midguts

**DOI:** 10.1371/journal.pntd.0014592

**Published:** 2026-07-31

**Authors:** Godfrey Nattoh, Philip M. Armstrong, Doug E. Brackney

**Affiliations:** Center for Vector Biology and Zoonotic Diseases, Department of Entomology, The Connecticut Agricultural Experiment Station, New Haven, Connecticut, United States of America; Beijing Children’s Hospital Capital Medical University, CHINA

## Abstract

Arthropod-borne viruses (arboviruses) such as dengue virus pose a significant and growing threat to human health worldwide. Maintained in a transmission cycle between arthropod vectors and vertebrate hosts, arboviruses experience strong bottlenecks during transmission which can drastically alter virus population composition and fitness. In vectors, severe population bottlenecks occur at the initial site of infection, the midgut, yet the specific factors driving this process remain unclear. To investigate these mechanisms, we need a better understanding of early infection events; however, traditional detection methods lack the necessary sensitivity. Recent advances in molecular signal amplification-based methods now make it possible to study these early stages in detail. In this study, we examined early dengue virus 2 (DENV-2) infection of *Ae. aegypti* midguts using multiple hybridization chain reaction techniques. We demonstrate that these techniques are much more sensitive than the traditional immunofluorescence assay and can reliably detect DENV-2 in mosquito midguts as early as 6 hours post infection. Further, we observed significant bottlenecks as only a handful of virions initiate infection of the midgut. The application of signal amplification strategies now enables critical assessment of the cellular and molecular interactions governing early infection events which could inform the development of novel interventions.

## Author summary

Arthropod-borne viruses (arboviruses), like dengue virus (DENV), pose a continuous and significant threat to global public health. DENV is maintained in a transmission cycle between *Aedes aegypti* mosquitoes and vertebrate hosts. Mosquitoes can acquire infection upon acquisition of an infectious blood meal; however, this is predicated on the viruses ability to successfully establish infection of the midgut. The mechanisms mediating this initial infection event are poorly understood and traditional methods for virus detection lack the sensitivity required to closely study these early events. In this study, we utilized hybridization chain reaction, a molecular signal amplification technique, to track early DENV-2 infections in *Aedes aegypti* midguts. We found that these methods were more sensitive than standard techniques, allowing us to reliably detect DENV-2 infection of the midgut within 6 hours following acquisition of an infectious blood meal. Crucially, our imaging revealed that only a handful of midgut cells are initially infected, suggesting that DENV-2 populations experience strong population bottlenecks during infection of mosquitoes. These high-sensitivity tools provide a framework for future studies aimed at identifying cellular factors mediating virus infection, the results from which could lead to the design of novel transmission blocking interventions.

## Introduction

Dengue virus serotypes 1–4 (DENV1–4) are transmitted to humans through the bite of infected female mosquitoes, and continue to pose a significant global health challenge, with 7.6 million cases reported by WHO in 2024 alone [1]. *Aedes aegypti* is the primary mosquito vector responsible for maintaining this transmission cycle, and its expansion into new geographical territories beyond tropical and sub-tropical ranges into more temperate climates have been linked to an increase in virus transmission [2–7]. In the absence of effective vaccines or therapeutics for many *Orthoflaviviruses* and other related mosquito-borne pathogens, vector control remains the most effective strategy against these pathogens [3,8]. Given that *Aedes aegypti* mosquito populations are developing increased resistance to pyrethroids and organophosphate insecticides, there is a need for novel control strategies [9]. Understanding the early events mediating arbovirus infection of mosquitoes will be critical to achieving this goal.

Vector competence is defined as the arthropod’s ability to acquire a pathogen, support its replication and subsequently transmit it to a susceptible host. Mosquitoes become infected upon ingestion of an infectious blood meal, but viruses must overcome multiple barriers within the mosquito for successful transmission to occur [10]. Initiating infection of the midgut epithelium is arguably the most critical barrier; however, little is known about the factors mediating these interactions. The midgut epithelium is composed of 5,000–10,000 cells, and upon ingestion of an infectious blood meal, a handful of cells become infected, representing a significant population bottleneck [11–13]. Interestingly, studies on intra-host genetic diversity have shown that the effective population size (*N*_*e*_), defined as the number of unique viral genomes infecting the midgut, is ~ 2–161 genomes for orthoflaviviruses [14,15]. Together these findings suggest that multiple genomes/ virus particles are infecting a limited number of midgut epithelial cells. The reasons for this apparent tropism are unclear. Elucidating the factors mediating early infection events will be essential to developing novel control strategies targeting critical processes within these systems.

Studying early infection events at the arbovirus-vector interface can be challenging because traditional detection assays (immunofluorescence assays (IFA) and fluorescently tagged viruses) are not sensitive enough to detect low abundance viral proteins or RNA. The advent of signal amplification technologies now makes this possible. These technologies adapt traditional techniques so that the substrate/ ligand interaction can be fluorescently amplified. In this study, we applied hybridization chain reaction (HCR) with immunofluorescence (HCR-IF), fluorescent *in situ* hybridization (HCR-FISH) and flow cytometry (HCR-FlowFISH) to examine DENV-2 infection kinetics in *Ae. aegypti* midguts at early timepoints post-infection (6, 12-, 18-, 24- and 72-hours post infection (hpi)) and compared their sensitivity to IFA. We demonstrate that all of the methods can reliably detect DENV-2 at 24 and 72 hpi, but that IFA was unable to detect infection at earlier timepoints whereas HCR-IF, HCR-FISH and HCR-FlowFISH could detect active DENV-2 infections as early as 6 hpi. We observed significant bottlenecks with only ~2–40 midgut epithelial cells initially infected and infection concentrating in the posterior portion of the midgut. Together, this data demonstrates that signal amplification technologies enable the detection of initial infection events in the mosquito midgut at biologically and physiologically relevant time points. Such capabilities now make it possible to identify the cells infected during the early stages of infection which will be critical to elucidating the factors mediating infection.

## Methodology

### Virus and cell culture

*Aedes albopictus* C6/36 cells were maintained in minimum essential medium containing 10% fetal bovine serum (FBS), 1X non-essential amino acids (Gibco, Grand Island, NY, 11140050), 1X antibiotic-antimycotic (Gibco, Grand Island, NY, 15240062), and sodium bicarbonate. Cells were grown to ~90% confluency in T25 flasks and infected using 100 µl DENV-2 virus stocks (125270/VENE93; GenBank: U91870). The virus was diluted in 1mL media and overlaid on cells and incubated at room temperature on a rocking platform for 1 hr before incubating the infected cells at 28°C with 5% CO_2_ for 5 days. The viral titers ranged from 4.15 × 10^6^ − 1.65 × 10^7^ FFU (focus forming units)/mL as determined by focus forming assays as previously described [16].

### Mosquito rearing and virus infection

*Ae*. *aegypti* Orlando strain (Orlando, FL; 1952) mosquitoes were reared at 27°C with a 14:10 light: dark cycle. Mosquitoes were maintained on 10% sugar and colony maintained by feeding females on defibrinated sheep’s blood. Infection studies were completed with 5–7 day old females. Mosquitoes were provided with a blood meal containing virus supernatant and defibrinated sheep’s blood (HemoStat Laboratories) at a 1:1 ratio and allowed to feed for ~45 min by means of a water-jacketed membrane feeder. Fully engorged females were cold anesthetized and sorted into 6 groups (n=~20) for each timepoint (6, 12, 18, 24, and 72 hpi). Exposed mosquitoes were maintained at 27°C with a 14:10 light: dark cycle until sample collection. Midgut tissues were dissected in 1x PBS and the residual blood was removed by making a small incision at the frontal section of the posterior midgut and blood gently removed with a dissection pin and forceps. Samples were washed four times with 1X PBS by pipetting before fixing in 4% paraformaldehyde overnight at 4°C.

### Hybridization chain reaction fluorescent *in-situ* hybridization (HCR-FISH)

Fixed midguts were dehydrated to remove fixative using a graded methanol series in 0.1% Tween 20 in 1X PBS (0.1% PBS-T) as follows: 25% methanol in 0.1% PBS-T for 10 min., followed by 50% methanol in 0.1% PBS-T for 10 min., 75% methanol in 0.1% PBS-T for 10 min., and 100% methanol overnight (ON) at -20°C. Tissues were then rehydrated through a reverse methanol/0.1% PBS-T gradient (75%, 50%, 25%, 0%) and washed three times in 0.1% PBS-T at 10-min. intervals. Subsequently, tissues were digested with 20 μg/mL proteinase K (Thermo Fisher, AM2548) diluted in 0.1% PBS-T for 30 min. at room temperature (RT), followed by two 10-min. washes in 0.1% PBS-T at RT. Subsequently, the DENV-2 antigenome RNA probe set (Molecular Instruments), generated using the whole genome DENV-2 VENE93 strain, was prepared at 8 μmol concentration in pre-warmed probe hybridization buffer (PHB). Samples were hybridized ON at 37°. Following hybridization, samples were washed 3X for 10 min. with probe wash buffer (Molecular Instruments) pre-warmed to 37°C, followed by two washes with 5X saline-sodium citrate (SSC) buffer (Invitrogen, 15557044) containing 0.1% PBS-T.

Fluorophore-conjugated hairpins Alexa 647-conjugated B4-h1 and B4-h2 (Molecular Instruments) were prepared separately by heating to 95°C for 90 s., then cooled to room temperature (RT) for 30 min. in darkness. Hairpins were added to the amplification buffer (Molecular Instruments) at 60 nM concentration each, aliquoted to samples, and incubated ON at RT in darkness. Unbound hairpins were removed with two washes of 5X SSC containing 0.1% PBS-T. Tissues were counterstained with 300 μM DAPI for 10 min., washed three times in 5X SSC with 0.1% PBS-T, and mounted on glass slides.

### Immunofluorescence assay (IFA)

To perform IFA, samples were removed from fixative, permeabilized and blocked with 0.1% Triton X-100 and 1% BSA in 1X PBS for 1 hr. on a shaker at RT. Samples were then incubated with anti-flavivirus envelope mouse antibody (4G2, 1:500; Novus, NBP2–52709) in blocking solution (0.1% Triton X-100, 1% BSA in 1X PBS) ON at 4°C. Subsequently, tissues were washed three times in blocking solution before incubation with secondary anti-mouse IgG-Alexa 555 antibody (1:500) diluted in blocking buffer for 2 hrs. on a shaker at RT. Samples were washed 3X in blocking solution, counterstained with 300 μM DAPI for 10 min. and washed 3X in 0.1% PBS-T and mounted on slides using Prolong Gold Antifade with 4’,6-diamidino-2-phenylindole (DAPI) (Thermo Fisher, P36931).

### Hybridization chain reaction immunohistochemistry *(HCR-IF)*

To perform HCR-IF, fixed midguts were dehydrated to remove fixative using a graded methanol series as described above. Subsequently, samples were incubated in anti-flavivirus envelope mouse antibody (4G2, 1:500) in blocking solution (0.1% Triton X-100, 1% BSA in 1X PBS) ON at 4°C. Upon washing off the unbound primary antibody, they were incubated with secondary donkey anti-mouse IgG antibody (1:500) modified with initiators (B4-H1 and H2; Molecular Instruments). Subsequently, serial washing steps and incubation with fluorophore-conjugated hairpins (Alexa 647-conjugated B4-h1 and B4-h2) was completed as described above. Unbound hairpins were removed, and samples were counterstained with DAPI before mounting on slides.

### Confocal laser scanning microscopy (CLSM) and image processing

Stained midgut mounted on slides were immediately visualized using confocal laser scanning microscopy (CLSM; Zeiss LSM 880 system, Germany) equipped with an HXP light source at the Yale Science Hill imaging facility. Images were visualized using emission wavelengths 561 nm (DENV-2 protein; IFA), 633 nm (DENV-2 protein – HCR-IF and DENV-2 RNA – HCR-FISH) and 405 nm (cell nucleus), and acquired using 20x objective with a 3172 x 3172-pixel resolution. Negative samples were used for standardization before acquiring all images. A 4X4 tiled images were generated to quantify the number of DENV-2 infection foci and the number of cells per infected midgut using ImageJ. To acquire an accurate estimate of the number of cells per foci we used the polygon selection tool to mark region of interest (ROI) depicting area of the foci based on channels 561 nm and/or 633 nm so that only the pixels within these areas were selected for analysis. The number of cells within a marked ROI was determined using channel 405 nm to avoid overestimation of infection intensities. Images were excluded from analysis if DENV-2 and cell nucleus staining signals were not clearly distinguishable. The number of foci in each midgut and the number of cells per foci were compared across timepoints. Foci and cell counts were graphically represented using GraphPad Prism 9 software package (GraphPad Software, San Diego, CA), and non-parametric Kruskal Wallis test undertaken to establish if differences between timepoints examined were statistically significant at p < 0.05.

### Hybridization chain reaction-flow cytometry-FISH (HCR-FlowFISH)

For each of the two independent experimental replicates, approximately 10 mosquito midguts were harvested for each timepoints at 6, 12, 18, 24, and 72 hpi, grouped in pools of five, fixed in 4% PFA at 4°C ON and subjected to the HCR-FISH protocol as described above. Subsequently, samples were washed 3X in 500 µL of 1x PBS for 10 min. The midguts were then transferred into a clean 1.5 mL microtube prior to cell dissociation as previously described [17]. Briefly, 300 µL of *Bacillus licheniformis* protease (10 mg/mL) dissociation buffer, which included Sf900III media (Gibco) and DNAse I (25 U/mL), was added to 10 midguts and triturated using a p1000 pipette at intervals of 15–20 min. for 120 min. During each interval, 120 µL of dissociated single cells were collected before replenishing with an equal volume of dissociation media. The collected cells were suspended in 25 mL of Sf900III + 5% FBS, kept on ice, and later centrifuged at 700xg for 10 min. at 4°C. The concentrated cells were then resuspended in 500 µL of Sf900III + 5% FBS, filtered through a 40 µm filter (PluriSelect), and counted using the Countess II Automated Cell Counter (Thermo Fisher Scientific). A detailed protocol for cell suspension preparation can be found at dx.doi.org/10.17504/protocols.io.j8nlke246l5r/v1.

### Flow cytometry analysis

The flow analysis of fluorescence signals on the DAPI and Alexa 647 channels was performed using the BD LSRFortessa Cell Analyzer (Becton, Dickinson and Company, BD Biosciences, San Jose, CA, USA), while cell sorting was executed on the BD FACSAria III instrument (Becton, Dickinson and Company, BD Biosciences, San Jose, CA, USA). For the flow analysis, cell nucleus was excited using a 405 nm laser, whereas Alexa 647 staining DENV-2 was excited with an APC laser. Unstained cells were included to establish threshold limits with a minimum number of events. The voltages for each channel were adjusted so that cells stained solely with DAPI and those stained only with Alexa 647 were positioned above the threshold scale. Tubes containing experimental samples were briefly vortexed for 20 s. prior to loading onto the instrument for acquisition at a flow rate of 12 µL/min to collect 10,000 events. Gating was performed on a forward and side scatter bivariate dot plot to eliminate cell debris. The gated cell populations were subsequently categorized based on APC emission to estimate DENV-2 infection across various timepoints and to evaluate the proportion of the infected cell population. For cell sorting, a 70 µm nozzle and 70 PSI were employed on the BD FACSAria III instrument to sort cells into a tube containing 1x PBS. The sorted cells were then centrifuged at 500xg for 5 minutes and examined under a confocal microscope.

## Results

### HCR-FISH and HCR-IF readily detect dengue virus infection of *Aedes aegypti* midguts

**Fig 1:**
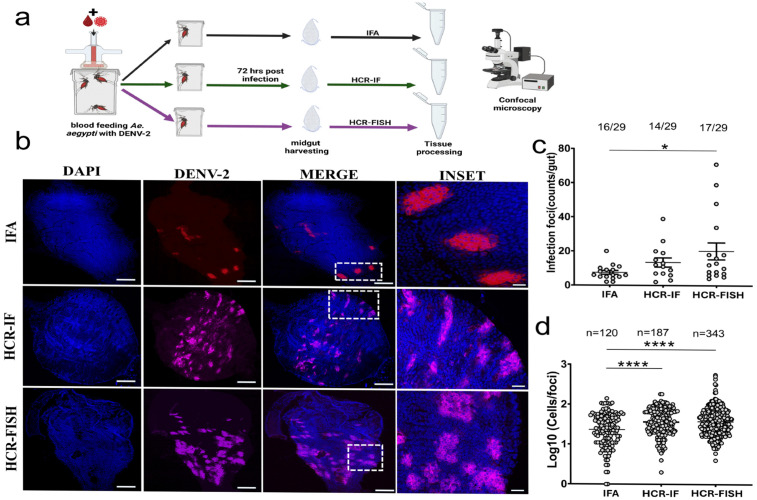
Hybridization chain reaction can reliably detect DENV-2 in *Aedes aegypti* midguts. a) Experimental design for infection study and midgut processing for HCR-FISH, HCR-IF, and IFA staining methods. *Created in BioRender,* https://BioRender.com/rh4adl9 b) Representative confocal microscopy images of DENV-2 infected mosquito midgut tissues. Top panel: IFA targeting DENV-2 E-glycoprotein (Alexa 555). Middle panel: HCR-IF targeting DENV-2 E-glycoprotein (Alexa 647). Bottom panel: HCR-FISH targeting DENV-2 antigenome (Alexa 647). Midguts were harvested 72 hpi, nuclei cells stained with DAPI (blue), while virus protein or nucleic acid stained either red and/or magenta. The inset shows a magnified view shown in the merged panel. Scale bars: inset = 50µm, other panels = 200µm. c) The number of infection foci in an individual midgut across the detection methods. d) The average number of cells per focus was obtained by dividing total infected cells in a midgut by the number of foci. Comparisons were made using Kruskal-Wallis test with Dunn’s multiple comparisons. *p  ≤  0.05, ****p  ≤  0.0001. Lines indicate mean  ±  standard error of the mean of the total foci or mean cells.

Early detection of infection events is necessary to identify the cellular and molecular factors mediating this process. In mosquitoes, arboviruses are known to initially infect only a few cells in the midgut following an infectious blood meal [6,13,18]. IFA is routinely used to visualize the kinetics of infection; however, this traditional approach lacks the sensitivity to detect low abundance viral antigens during early timepoints. Conversely, PCR-based approaches rely on destroying the tissue and cannot easily differentiate between active infections vs. viral particles remaining in the blood bolus. Therefore, because signal amplification techniques can, in theory, detect a single molecule, we tested if this approach could be used to detect DENV-2 in the midguts of *Aedes aegypti*. We evaluated sensitivity of HCR targeting both DENV-2 protein (HCR-IF) and genomic RNA (HCR-FISH) and compared it with IFA in mosquito midguts 72 hpi (Fig 1a, unstained midguts included in S1 Fig). DENV-2 infection was readily observed forming distinct foci across all detection strategies evaluated (Fig 1b), corroborating previous observation [6]. We observed a significant difference in the number of foci detected by the three methods (Kruskal-Walli’s test, *H*(3) = 6.6, *p* = 0.0374; Fig 1c). Pairwise comparisons confirmed significant differences between IFA and HCR-FISH (*p* = 0.047). However, the abundance of infection foci did not differ between HCR-IF and IFA or HCR-FISH (Fig 1c). We also observed a significant difference in the number of cells per foci (Kruskal-Walli’s test, *H*(3) = 26.35, *p <* 0.0001). Pairwise comparisons confirmed significant differences in the number of cells per foci between IFA and HCR-FISH or (*p <* 0.0001; Fig 1d). These results demonstrate that HCR-based methods reliably detect both DENV-2 protein and RNA during DENV-2 infection of mosquito midguts.

### HCR detection at early timepoints reveals DENV-2 population bottlenecks during midgut infection

**Fig 2:**
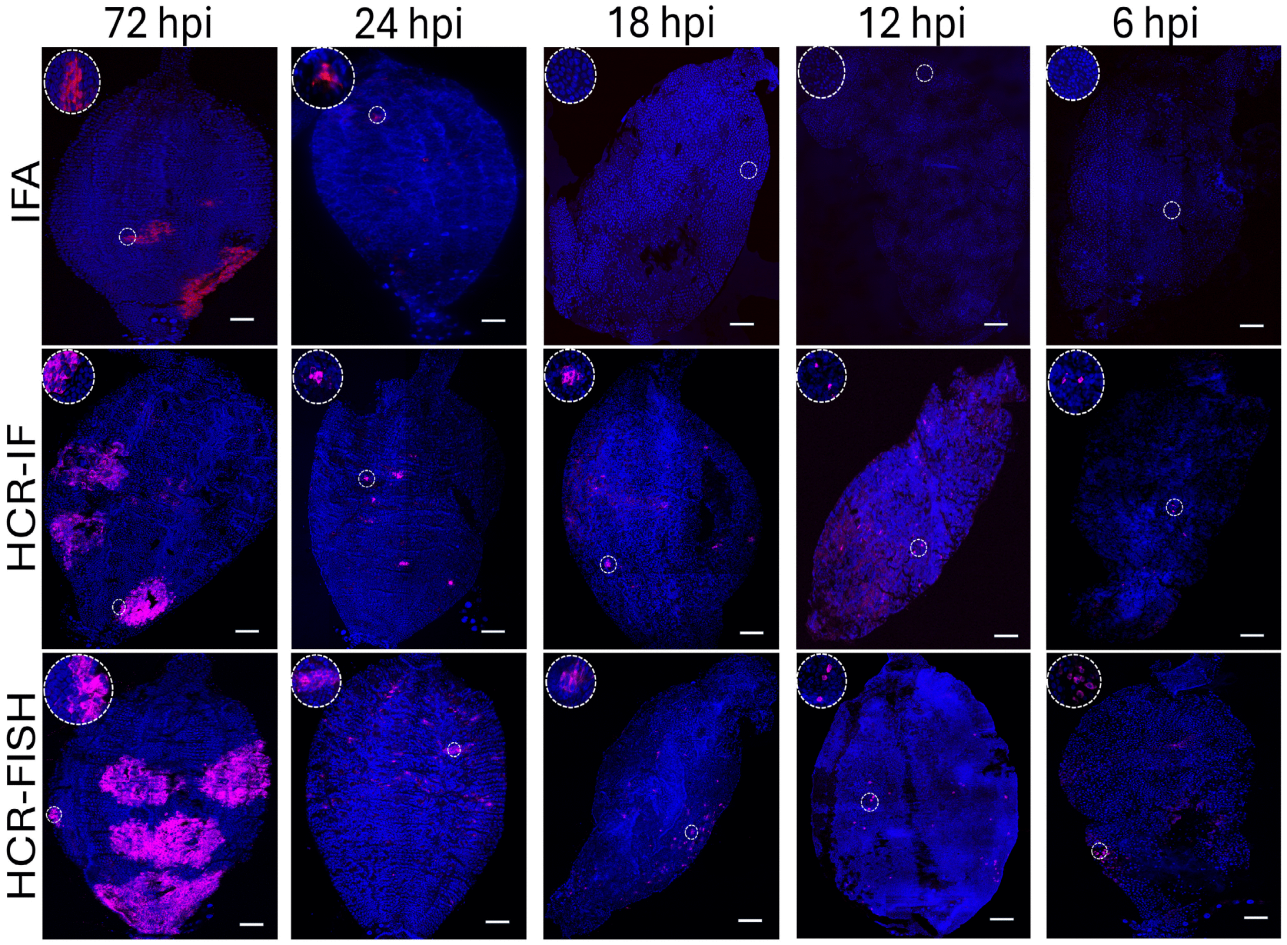
Detection of DENV-2 RNA in *Aedes aegypti* midguts as early as 6 hours post infection. Representative confocal microscopy images showing time-course analysis of DENV-2 infection in mosquito midgut tissues. Representative images obtained at 6, 12, 18, 24, and 72 hpi. Top panel: IFA targeting DENV-2 E-glycoprotein. Middle panel: HCR-IF targeting DENV-2 E-glycoprotein. Bottom panel: HCR-FISH targeting DENV-2 antigenome. Scale bars: = 200µm.

Having demonstrated the utility of HCR in our system, we subsequently assessed the sensitivity of each of the three approaches at multiple early timepoints post infection (i.e., 6, 12, 18, 24, and 72 hpi). As expected, IFA could readily detect DENV-2 antigen at 24 and 72 hpi, the foci were well defined and individual infected cells could be visualized at 24 hpi (Figs 2; S1). At 24 hpi, HCR-based techniques revealed significantly higher mean foci than IFA (Kruskal-Walli’s test, *H*(3) = 9.331, *p* = 0.0094; S2 Fig). Pairwise comparisons confirmed significant differences between IFA and HCR-FISH (*p* = 0.0077), though IFA and HCR-IF were not significantly different. IFA failed to detect DENV-2 antigen at the earlier timepoints (Fig 2; top panel), whereas both HCR-IF and HCR-FISH detected viral antigen and antigenomic RNA at all timepoints tested (Fig 2; middle and bottom panels). Further, while single cell infections were observed at 24 hpi by IFA, clear multi-cellular foci were evident by both HCR approaches at this timepoint (S1 Fig). This demonstrates that DENV-2, and presumably other arboviruses, are establishing infection, replicating and infecting neighboring cells within the first 24 hpi. Interestingly, we observed clusters of infected cells by both HCR-FISH and HCR-IF at very early timepoints post infection (i.e., 6 and 12 hpi) (Figs 2; S1). Because these clusters exist before the virus has adequate enough time to replicate, they most likely represent independent infection events and not expanding foci. Such observations have been observed in other mosquito-arbovirus systems [12,13].

**Fig 3:**
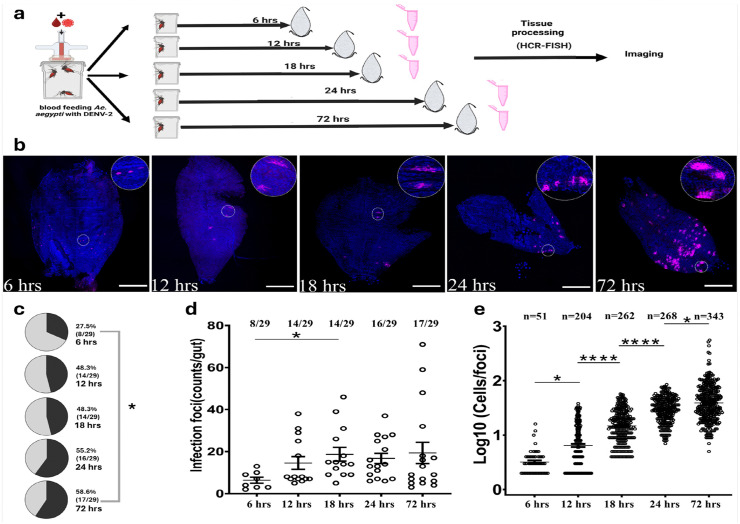
Population bottlenecks associated with DENV-2 infection of mosquito midguts. a) Experimental scheme for time-course imaging of DENV-2 infection outcome in *Ae. aegypti* midgut using HCR-FISH staining. *Created in BioRender,* https://BioRender.com/3xya7s0. b) Representative confocal microscopy images of DENV-2 infected mosquito midgut tissues across all timepoints investigated (i.e., 6 hpi vs 12 hpi vs 18 hpi vs 24 hpi vs 72 hpi). The inset shows a magnified view of the circled portion. Nuclei cells stained with DAPI (blue), while virus RNA-stained magenta (Alexa 647). Scale bars = 200µm. **c)** Midgut infection prevalence across timepoints assessed relative to 6 hpi by Fisher’s exact test. **d**)Time course of DENV-2 infection foci per infected midgut. The graph shows abundance of infection foci over sampled timepoints. Statistical significance of differences in number of infection foci was assayed using Kruskal-Wallis test with Bonferroni’s multiple comparisons and shown in the figure (*p  ≤  0.05). Lines indicate mean  ±  standard error of foci abundance. **e**) Time course of DENV-2 mean cells per infected foci. The graph shows the mean  ±  SEM abundance of cell infected over sampled timepoints. The total number of foci counted is indicated. Statistical significance of differences in infected cells was assayed using Kruskal-Wallis test with Dunn’s multiple comparisons uncorrected. Different number represents statistical significance (p < 0.0001).

**Fig 4:**
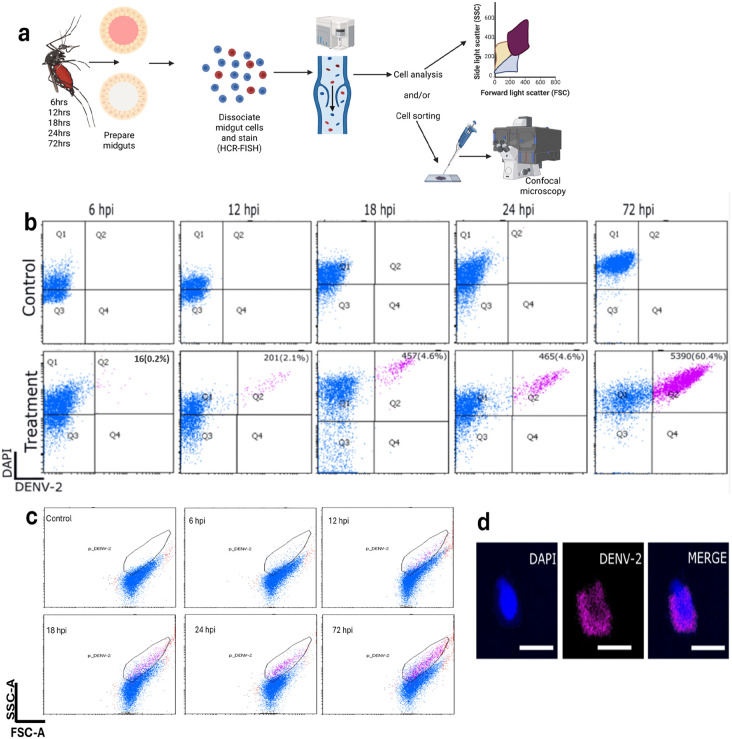
Temporal analysis of DENV-2 infection in mosquito midguts by flow cytometry. a) Experimental scheme for time-course detection of DENV-2 infection outcome in *Ae. aegypti* midgut using HCR-FlowFISH method *Created in BioRender,* https://BioRender.com/wt9ree7. b) Bivariate dot plots of the nucleus (DAPI, y-axis) versus DENV-2 (Alexa 647, x-axis) showing the proportion of cells infected at various timepoints examined (6, 12, 18, 24, & 72 hpi). Proportions represented are of cells located in the second quadrant (Q2). c) Representative flow cytometry dot plots illustrating the gating strategy used to isolate specific cell populations (circled in a polygon). Identical gating was applied across all monitored timepoints to ensure longitudinal consistency in population analysis. The proportion of infected cells are as shown in brackets. d) Representative confocal microscopy images of cell populations isolated via fluorescence-activated cell sorting (FACS). Scale bars = 20µm.

We further examined and quantified DENV-2 infection kinetics across this time series using HCR-FISH (Fig 3a). The initiation of DENV-2 infection was noted in individual cells and clusters (Fig 3b **6 hpi panel**), with the foci enlarging as infection spreads laterally to adjacent cells at subsequent timepoints (Fig 3b **18–72 hpi panels**). We assessed the prevalence of infection at each timepoint and noted a significant difference in DENV-2 infection rates between the earliest timepoint (6 hpi (8/29; 27.5%)) and the last timepoint (72 hpi (17/29; 58.6%)) (p = 0.033) by Fisher’s exact test. There were no significant differences in prevalence between any of the other timepoints (12 hpi (16/29; 55.2%)), (18 hpi (14/29; 48.3%)), and (24 hpi (16/29; 55.2%)) (Fig 3c). Subsequently, we quantified the number of foci/ infected midgut and found that there was no significant difference between the number of foci across time points (Kruskal-Walli’s test, *H*(5) = 9.453, *p* = 0.051, but pairwise comparison confirmed that midguts at 6 hpi had significantly fewer foci than 18 hpi (p = 0.036; Fig 3d). Together, this data suggests that assaying for infection at very early timepoints (i.e., 6 hpi) may not fully identify all foci that will form and/ or all midguts that will eventually become infected. In addition, we also enumerated the number of infected cells per infected midgut for each timepoint using a semi-quantitative microscopy-based ImageJ approach. We observed an exponential increase in infected cells from 6 hpi to 12 hpi (*p* = 0.015), 12 hpi through 24 hpi (Kruskal-Walli’s test, *H*(3) = 149.1, *p* < 0.0001) and 24 hpi to 72 hpi (*p* = 0.039 (Fig 3e). Subsequently, we utilized HCR-FlowFISH to validate these findings (Fig 4a). For each timepoint, mosquito midguts were dissected from five individuals and pooled together for processing and analysis. As expected, a small proportion of cells were initially infected at 6 hpi (n = 16, ~ 0.2%). The proportion increased at subsequent timepoints to 201 cells (2.1% at 12 hpi), 457 and 465 cells (18 and 24 hpi respectively, 4.6%), and 5,390 infected cells at 72 hpi (~60.4%) (Figs 4b, S3). Interestingly, the infected cell populations appeared to form a cluster of closely related cells which could be suggestive that specific cell populations are more likely to support viral replication. However, this could also be an artifact of infection such that infected cells clustered together simply because they were infected (Fig 4c). To confirm the HCR-FlowFISH results, infected cells were sorted and analyzed microscopically to show the presence of DENV-2 RNA (Fig 4d).

### DENV-2 infection of midgut cells is compartmentalized

**Fig 5:**
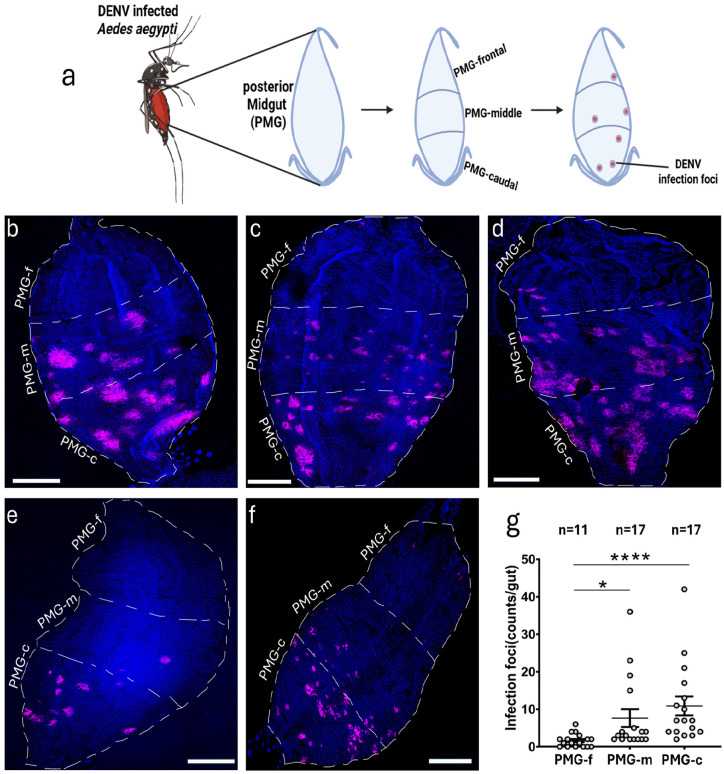
Spatial distribution of DENV-2 infection foci across midgut epithelium. a) Illustration of compartments of *Ae. aegypti* posterior midgut (PMG) infected with DENV-2 into frontal (PMG-f), middle (PMG-m), and caudal (PMG-c) sections *Created in BioRender,* https://BioRender.com/08mbex3. b-f) Representative confocal microscopy images of *Ae. aegypti* midgut tissue at 72 hpi with DENV-2, showing HCR-FISH targeting DENV-2 antigenome (Magenta), cell nuclei (DAPI, blue). g) The graph shows abundance of DENV-2 infection foci across different compartments of the infected posterior midguts (n = 17). Statistical significance of differences in number of infection foci was assayed using Kruskal-Wallis test with Dunn’s multiple comparisons and shown in the figure (*p  ≤  0.05, ****p  ≤  0.0001). Scale bars represent 200 μm.

Previous studies show regional patterning within the midgut, along with significant transcriptional variations across sections [19]. This observation could imply the potential for differences in anatomical and physiological responses, which could also influence the outcome of infection with arboviruses such as DENV. We examined and quantified DENV-2 infection across sections of frontal posterior midgut (PMG-f), middle (PMG-m), and caudal (PMG-c) (Fig 5a). We observed distinct significant distribution of DENV-2 infection foci across midgut compartments (Kruskal-Walli’s test, *H*(3) = 21.7, *p*=<0.0001). This infection patterning appeared more pronounced in the middle (PMG-m; p = 0.0142) and caudal (PMG-c; *p* < 0.0001) relative to the frontal segment of the posterior midgut (PMG-f; Fig 5b). The observations were consistent across all detection methods investigated with infection appearing to readily spread in the same pattern (S4 Fig). If virus infection is more likely to begin and readily spread in the distal section of the posterior midgut, it may suggest the presence of host factors necessitating infection.

## Discussion

The ability of arboviruses to infect the mosquito midgut represents a critical determinant of transmission and a potential target for novel interventions; however, the mechanisms underlying infection initiation remain poorly understood. This is partially due to the fact that we do not have good tools to examine the early infection events as traditional virus detection methods lack the sensitivity required to detect low abundance viral antigens during the early stages of infection [6,20]. For instance, strategies such as IFA and recombinant fluorescent viruses require multiple rounds of replication in order to generate detectable levels of either viral protein or a fluorescent reporter, respectively [6,20]. Consequently, by the time these techniques can be utilized to interrogate virus-midgut interactions, the virus has already begun to spread to neighboring cells. These methodological limitations have made it difficult to examine the initial virus-cell interactions mediating infection. This is particularly relevant because others have demonstrated that infection is initiated in a limited number of cells in a non-random manner [13,18]. The advent of molecular signal amplification-based strategies now makes it possible to detect low abundance molecules. HCR can be coupled with immunofluorescence (HCR-IF) and RNA fluorescence *in situ* hybridization (HCR-FISH) to detect low abundance protein and RNA molecules, respectively and flow cytometry and cell sorting (HCR-FlowFISH) to identify and collect cells containing molecules of interest [21]. We employed imaging (HCR-IF and HCR-FISH) and flow cytometry (HCR-FlowFISH) to investigate DENV2 infection of *Aedes aegypti* midguts at early timepoints (6, 12, 18, 24, and 72 hpi) and demonstrate that these techniques can be used to confidently identify virus infected cells as early as 6 hpi and show that DENV-2 genomic material can be detected in neighboring cells as early as 12 hpi. In contrast, we were unable to detect DENV-2 infected cells at 6, 12, or 18 hpi when using traditional IFA. Together, these data demonstrate the utility of signal amplification techniques for studying early events during arbovirus infection of mosquitoes.

The peritrophic matrix 1 (PM1) is a blood meal inducible semi-permeable matrix composed of glycoproteins and chitin that begins to form in the midgut 4–6 hours post-blood feeding reaching maturity by 12 hrs [22]. It functions to modulate blood meal digestion and protect the midgut epithelium against mechanical and chemical damage as well as ingested pathogens [23,24]. In this study, we observed reduced infection prevalence and fewer infection foci at 6 hpi compared with all of the other timepoints. A possible explanation for these observations is that DENV-2 is able to overcome the formation of the PM1 and continue initiating new infections beyond 6 hpi; however, we find this scenario unlikely because others have found that the PM1 serves as an important barrier to arbovirus infection [25,26]. Furthermore, as the digestive process progresses, virus particles are purged from the system through diuresis and are exposed to an increasing concentration of proteolytic enzymes. A more likely explanation is that DENV-2 infects the midgut asynchronously within the first 6 hrs. such that some virus particles initiate infection of the midgut epithelium rapidly while others may not initiate infection until later timepoints. Consequently, infection events occurring <1 hpi may be more readily detectable at 6 hpi than other events that were initiated >4 hpi. This could explain why we observed increased infection rates and foci at 12 hpi compared to 6 hpi but not later timepoints (i.e., 12 vs. 18 or 24 hpi). Nevertheless, this data demonstrates that DENV-2 infects *Ae. aegypti* within 12 hours of a blood meal, and likely at much earlier timepoints.

In this study, we observed that DENV-2 infection of the mosquito midgut is initiated in a limited number of foci (e.g., 2–13 foci at 6 hpi and 2–32 foci at 12 hpi), a similar trend was reported for West Nile virus (WNV) and Venezuelan equine encephalitis virus (VEEV) [13,18]. Together, these findings demonstrate that the mosquito midgut infection is accompanied by a strong population bottleneck. It is surprising that so few cells were initially infected given that the *Ae. aegypti* midgut is composed of 5,000–10,000 cells and, during these experiments, mosquitoes ingested ~ 5x10^3^ virus particles [27,28]. The reasons for this observation are unclear. It is possible that infection is a stochastic event dictated by biochemical (i.e., exposure to digestive enzymes) and biophysical (i.e., diuresis) forces and the sheer chance that a 50 nm virus particle encounters a susceptible midgut cell while suspended in a sea of blood (~2–3 µl). However, it was previously observed that VEEV infection of *Aedes taeniorhynchus* occurs non-randomly [13]. Using single-round infectious VEEV particles expressing green fluorescent protein (VEEV-GFP) and cherry fluorescent protein (VEEV-CFP) at equal concentrations, it was found that one-third of all infected cells exhibited dual infection [13]. Similar results have been found when estimating the effective population size of orthoflaviviruses during horizontal transmission to mosquitoes. These studies found that between 2–161 genomes originating from the blood meal can be found in the midgut [14,15]. These results are highly probabilistically unlikely if infection occurred entirely at random and suggest other factors may be important in mediating midgut infection. One such factor could be the distribution and abundance of arbovirus receptors. Numerous studies have identified mosquito midgut proteins that interact with arboviruses, but no true receptor has yet to be identified [27,29–32]. The existence of bona fide receptors with heterogeneous distributions across the midgut epithelium could account for these observations. Alternatively, this data could reflect a tropism by arboviruses for a specific cell type or cellular state. There are four primary cell types in the mosquito midgut - intestinal stem cells, enteroblasts, enterocytes and enteroendocrine cells [11,17,33]. In addition, single-cell sequencing has found that the enterocytes and enteroendocrine cells can be further divided into subpopulations with unique transcriptional profiles which could be potentially further subdivided under different physiological/ metabolic states (i.e., blood feeding and different stages of digestion) although this has not been confirmed [11,17,33]. It should be noted that virus receptors and/ or cell types are likely not the only factors mediating arbovirus infection of midgut epithelial cells. Our data demonstrates that HCR can be a powerful tool to examine the early events of infection which could be used in conjunction with other advanced molecular techniques to help elucidate the role of these factors in mediating arbovirus infection of the midgut epithelium in future studies

We observed that DENV-2 preferentially initiates infection of the caudal section of the posterior midgut. The reasons for this are unclear, but there are a number of factors which could account for this observation. Following blood meal acquisition, mosquitoes undergo diuresis, which involves rapid expulsion of the excess fluid component from the ingested blood [34]. This process likely concentrates viral particles in the caudal region of the posterior midgut region, thereby increasing the probability of viral particles encountering epithelial cells and initiating infection. If this hypothesis were correct, we would expect that all arboviruses would have a spatial preference for the caudal region of the posterior midgut; however, previous studies found that Sindbis virus (SINV; *Alphavirus*; *Togaviridae*) has a more centralized midgut infection pattern in *Ae. aegypti* [35,36]. It is possible that the caudal region of the posterior midgut has a higher abundance of DENV-2 susceptible cells, but that SINV susceptible cells are more abundant in the central region. Such differences could account for the observed discrepancy. Alternatively, the posterior mosquito midgut may be regionally compartmentalized as has been described for the gut of *Drosophila melanogaster* [19,37]. Such compartmentalization could result in morphological, molecular, and/or physiological differences impacting the ability of DENV-2 to establish infection in specific regions. This could be further confounded by other non-mutually exclusive factors including Brownian motion occurring within the ingested blood bolus and heterogeneity in peritrophic matrix permeability [26,38,39]. An understanding of how these factors interact to contribute to spatial restriction of virus infection may unravel key insights that could be modelled to further explain virus-vector interactions.

This work provides important insights into *Ae. aegypti* midgut infection dynamics by demonstrating that DENV-2 initiate infection within the first 6 hbi within only small number of cells and that infection preferentially occurs in the caudal section of the posterior midgut. The use of HCR, a signal amplification strategy, enabled detection of low abundant molecule and showed higher detection sensitivity of viral infection at 6–18 hpi, a capability that IFA lacked. These early timepoints post infection could provide a window of opportunity for elucidating the factors mediating infection. To the best of our knowledge, this is the first study documenting midgut infection with DENV2 at these early points of infection. Future identification and validation of host factors critical for viral infection could inform the development of transmission blocking strategies aimed at disrupting transmission of arboviral infections.

## Supporting information

S1 Fig Hybridization chain reaction negative controls. Confocal microscopy images of *Aedes aegypti* midgut tissue at 6, 12, 18, and 72 hpi showing representative negative control samples. Scale bars represent 100 μm (zoomed areas are circled inset).
(TIF)

S2 Fig Comparison of detection strategies where all methods identified DENV-2 infection at 24 hpi. Data represent mean ± SEM of DENV-2 infection foci per infected midgut. Statistical comparisons of infection foci between detection methods were undertaken using Kruskal-Wallis test with Dunn’s multiple comparisons. Sample size and infection prevalence are indicated above each column.
(TIF)

S3 Fig Flow cytometric histograms of DENV-2 infected *Ae. aegypti* midgut cell suspensions. The bars represent the proportion of positive cells at 6, 12, 18, 24, and 72 hpi. Uninfected midgut was included as control.
(TIF)

S4 Fig Spatiotemporal distribution of DENV-2 in *Aedes aegypti* midgut epithelium. Confocal microscopy images of *Aedes aegypti* midgut tissue at 24, 72, and 96 hpi with DENV-2, showing IFA targeting DENV-2 E-glycoprotein (red -Top Panel), cell nuclei (DAPI, blue), HCR-IF targeting DENV-2 E-glycoprotein (Magenta – Middle Panel), and HCR-FISH targeting DENV-2 antigenome (Magenta – Bottom Panel). Images reveal a non-uniform viral distribution with distinct foci of heavily infected cells occurring on the caudal region. Scale bars represent 100 μm (zoomed areas are circled inset).
(TIF)

S1 File Source data for figures 1c, 1d, 3c, 3d, 3e, 5g and S2 Fig. (XLSX)
