# Supplementary material for: Population bottlenecks and temporal constraints shape dengue virus infection of Aedes aegypti midguts
Source: PLoS Negl Trop Dis. 2026 Jul 31;20(7):e0014592. doi: 10.1371/journal.pntd.0014592 (PMC13426913; doi:10.1371/journal.pntd.0014592)
Supplement: Attachment — Submitted filename: Response to reviewers.docx [file pntd.0014592.s007.docx]

Response to Reviewer’s Comments PNTD-D-26-00483

We would like to thank the reviewers for their time and constructive feedback on our manuscript. Below, we provide a detailed response to each of their comments.

**Reviewer 1:**

-I may be wrong but I am not fully aware how this method will help in the control of dengue.

***Response: This method is not intended to help with the control of dengue virus directly, but rather a technological advancement that can be used to better understand virus-mosquito interactions. The knowledge from such studies could be used to develop novel control interventions. We believe this is made clear throughout the manuscript and no changes have been made.***

-It is a new method to determine the dengue virus at a very early stage. I have nothing against it. But *Ae. aegypti* is a known as a good dengue vector. How is this method going to be useful in the future. Perhaps this needs to be explained.

***Response: The application of signal amplification methods enabled the identification of early infection events. Understanding early infection events will enable researchers to begin to untangle the mechanisms of infection. We noted only a few infection events per midgut, suggesting that strategies targeting these rare events could render Aedes aegypti refractory to infection. We believe this was made clear in the manuscript and no changes have been made.***

- This is something that I am not convinced. I may be wrong but I feel there should be more explanation on why this study was carried out.

***Response: The purpose of this study was to compare the conventional viral detection method (IFA) with emerging signal amplification technologies (IF-HCR, HCR-FISH, HCR-FLOW). The goal was to address the shortcomings of traditional assays in detecting and quantifying the initial viral infection events in mosquito vectors, which are hindered by sensitivity limitations. By demonstrating that these new techniques can identify viruses at early time points (6-12 hours post-infection), researchers can utilize these methods to uncover the mosquito factors that viruses exploit to initiate midgut infections, potentially informing the development of new strategies that target these host factors. We feel this was made clear throughout the manuscript and no changes have been made.***

- It is a very interesting study and details have been provided. However, I have a few questions. *Ae. aegypti* is a well know vector for dengue virus. Here you have used a special method to detect the virus at 6hr after ingestion of blood.
How will this method be used stop dengue transmission? This needs to be mentioned in more details.

***Response: The use of signal amplification strategies has revealed that the onset of viral replication is orchestrated by a limited number of cells, suggesting that only a few midgut epithelial cells are prone to initial viral infection. This discovery holds significance for future research and may inform efforts to identify the traits of these cells, potentially leading to the development of drugs aimed at blocking viral transmission.***

- Yes some scientists use RT-PCR to detect dengue virus in wild caught mosquitoes and they have reported that it could be a method for dengue control. However, we know that the control programme may find it difficult to carry out RT-PCR.
Now it has been established that that the NS1 antigen test kit can detect dengue virus in mosquitoes and this is a simple method that can be used by the control programme. It has also been established that there is a correlation between infected mosquitoes and DF. Besides once an infected mosquito is found dengue will occur lag of 1 to 2 weeks. Thus, action can be taken before cases are reported. Thus, can you please explain how the method that you all have developed can be used to control an outbreak.

***Response: The approach utilized in this paper is not intended to be used for diagnostics in field or surveillance settings. This is a laboratory-based detection method to help interrogate early infection events. Coupled with other experimental approaches this could be used to uncover factors important in mediating virus infection of the mosquito which could then be exploited by novel control strategies to halt transmission.***

- *Ae. aegypti* mosquitoes will lay eggs 48 to 72 hours after a blood meal. It is perhaps during this time that the mosquitoes can be easily captured.
I have nothing against the methods that you all have described. I am only interested in knowing how the transmission blocking strategy can be used?

***Response: Please refer to the clarifications above.***

**Reviewer 2:**

- Results are good. Also I might have missed it... In the title it says "population bottlenecks AND temporal constraints shape dengue virus infection... what exactly is the population bottleneck you speak of? Is that in relation to the low number of cells infected in the midgut via your more precise method? I'm not exactly sure how you can jump to a conclusion about population bottlenecks with a single strain of *Aedes aegypti* maintained in a lab colony since 1952 - that colony IS a bottleneck.

***Response: We demonstrate that although a mosquito may ingest over 1000 viral particles, each having the potential to infect one of the approximately 5000 midgut epithelial cells, only a limited number of these ingested virions are able to successfully initiate an infection in the midgut epithelium. This represents a significant population bottleneck for the virus, not the mosquito so it is unclear the relevance of the bottlenecks associated with mosquito colonization.***

-Conclusions are good. However, I am curious, are there any concerns regarding this novel technique in relation to cross reactivity? Can you be certain it's a dengue virus and not something else, like an enteric viral infection found in the colony? This can occasionally even happen with PCR and ELISA assays - cross reactivity.

***Response: The techniques in question are probe-based. The probes are developed from the DENV-2 sequence, which enhances their specificity and significantly reduces the chance of cross-reactivity.***

- I only had two concerns... the rest of the paper was well written. The discussion section and the pros in the use of this technique to elucidate viral infection time in support of vector competence studies was clever. I too am curious now why only a couple cells appear to become infected in the midgut when there are loads of virus and apparently plenty of cells to infect. Not sure about the hypothesis that viruses infect the cells that are concentrated in the caudal region to allow water to be filtered though... then that would propose mosquitoes that do not feed to repletion and therefore do not concentrate cells/virus in the caudal region of the posterior midgut have a lower propensity of infection... food for thought.

***Response: The exact reason for the initial infection of only a few cells is not known. Our current hypothesis is that a subpopulation of midgut epithelial cells are susceptible to infection, thereby reducing the total number of infectable cells. Having established HCR and demonstrated its utility in identifying early infection events, we can begin to test this hypothesis. The question if the size of the blood meal impacts the spatial distribution of infection events is an interesting one. Unfortunately, we only examined mosquitoes that had acquired a full blood meal and, therefore, cannot directly speak to whether or not non-replete mosquitoes would have different infection patterns.***

**Reviewer 3:**

- Objectives & Hypotheses: The objectives are clearly articulated. The implicit hypothesis that signal-amplification technologies can reveal early, hidden viral dynamics and population bottlenecks is systematically tested.

Study Design: The comparative framework evaluating HCR-IF, HCR-FISH, and HCR-FlowFISH directly against traditional IFA is exceptionally robust and logically sound.

Sample Population: The choice of Aedes aegypti (Orlando strain) and DENV-2 (VENE93 strain) is standard and appropriate for vector competence evaluations. Statistical Power & Analysis: Non-parametric statistical approaches (Kruskal-Wallis with post-hoc multiple comparisons and Fisher’s exact test) are well-selected and applied properly given the distribution patterns of viral infection foci.
Ethical Requirements: The research utilizes commercially sourced defibrinated sheep's blood via artificial membrane feeders for mosquito colony maintenance and experimental infections. There are no human subjects or live vertebrate animal ethical concerns.
Methodological Clarification Required: Under the "Hybridization Chain Reaction-Flow Cytometry-FISH" section, the authors state that midguts were grouped in pools of five. Please explicitly declare how many independent pools (biological replicates) were run per timepoint to generate the scatter graphs and statistical estimates.

***Response: Thank you for the overall positive assessment of our work. We have amended our Materials and Methods sections to better explain our replicate and pooling strategy. It reads: For each of the two independent experimental replicates, approximately 10 mosquito midguts were harvested per timepoint (6, 12, 18, 24, and 72 hpi). Midguts were grouped in pools of five, fixed in 4% PFA at 4°C overnight (ON), and subjected to the HCR-FISH protocol as described above.***

- Alignment with Plan: The presented results align cleanly with the stated comparative and kinetic objectives.
Clarity of Presentation: The results are highly readable, well-structured, and explicitly present both focus-forming counts and single-cell percentages.
Figure Quality: The schematic panels generated via BioRender clarify experimental workflows beautifully. Confocal micrographs present sharp spatial resolution, and the inset windows are exceptionally helpful for visualizing low-abundance signals.

***Response: We thank the reviewer for this assessment.***

- Data Support: The conclusions regarding a severe early midgut bottleneck and asynchronous viral entry window are strongly supported by the statistical divergence between HCR tools and IFA.
Limitations Addressed: The authors properly acknowledge the challenges of distinguishing initial multi-virion clusters from expanding lateral foci at ultra-early intervals (6–12 hpi).
Public Health & Field Advancement: The manuscript emphasizes that identifying early cellular interactions can assist in developing targeted transmission-blocking strategies, establishing solid translational relevance.

***Response: We thank the reviewer for this assessment.***

-1. Data Availability Statement: In the metadata table, the authors state: "All images were collected and stored by the corresponding author and can be accessed upon request."
Correction: This is non-compliant with PLOS Data Policies. Authors are required to make all minimal underlying data fully available without restriction. The raw numerical values behind graphs (Figures 1, 3, 4, and 5) must be uploaded as Supporting Information spreadsheets or deposited into a public repository (e.g., Dryad, Zenodo, or Figshare), and a repository accession link/DOI must be provided prior to final acceptance.
2. Terminology Consistency: On pages 19 and 20, the manuscript alternates somewhat inconsistently between using "hpi" (hours post-infection) and "hpbf" (hours post-blood feeding). Correction: Ensure standard, unified use of terminology throughout the text. If the authors view "hpi" and "hpbf" as interchangeable in this experimental layout, a single defining sentence should declare this early in the text to avoid reader confusion

***Response: To point 1, in our resubmission we have uploaded a spreadsheet with all of the raw data in an Excel spreadsheet.***

***Thank you for recognizing the inconsistencies in our use of hpi and hpbf. We introduced hpbf in the discussion section about the peritrophic matrix specifically because some of the cited work was done in the absence of virus infection. Where appropriate we have left as hpbf, but for all other instances in the presence of infection we have used the acronym hpi.***

- This manuscript addresses a significant and long-standing limitation in the field of arbovirology: the inability of traditional assays to detect and quantify initial viral infection events in mosquito vectors due to sensitivity constraints. By implementing hybridization chain reaction (HCR) coupled with immunofluorescence (HCR-IF), fluorescence in situ hybridization (HCR-FISH), and flow cytometry (HCR-FlowFISH), the authors successfully track dengue virus serotype 2 (DENV-2) kinetics in Aedes aegypti midguts at ultra-early timepoints (6, 12, 18, and 24 hours post-infection). The study provides clear empirical evidence of severe population bottlenecks, showing that only a minute fraction of ingested virions successfully initiate midgut epithelial infection. Furthermore, it identifies a strong spatial preference for infection initialization in the caudal section of the posterior midgut.
Strengths:
Methodological Innovation: The application of HCR signal amplification to the mosquito midgut provides an outstanding, highly sensitive alternative to classical immunofluorescence assays (IFA) and destructive PCR methods. Biological Insight: Capturing active replication events as early as 6 to 12 hours post-infection shifts our understanding of early arboviral intra-vector dynamics.
Experimental Rigor: The combination of confocal imaging quantification alongside single-cell dissociation and flow cytometry validation (HCR-FlowFISH) offers excellent data triangulation.
Weaknesses:
Data Availability Policy Violation: The current data availability statement ("accessible upon request") directly violates PLOS repository guidelines.
Flow Cytometry Sample Clarification: The replication layout and exact biological sample configuration for the flow cytometry pools require explicit clarification in the text

***Response: A description of sample size and experimental replicate has been included in the Flow Cytometry section.***
