# Supplementary material for: Population bottlenecks and temporal constraints shape dengue virus infection of Aedes aegypti midguts
Source: PLoS Negl Trop Dis. 2026 Jul 31;20(7):e0014592. doi: 10.1371/journal.pntd.0014592 (PMC13426913; doi:10.1371/journal.pntd.0014592)
Supplement: Peer review history

# Peer review: Decision Letter 0

**DOI:** 10.1371/journal.pntd.0014592.r001

**Authors:** Abdallah Samy, Ran Wang

16 Jun 2026

PNTD-D-26-00483

Population bottlenecks and temporal constraints shape dengue virus infection of Aedes aegypti midguts

PLOS Neglected Tropical Diseases

Dear Dr. Brackney,

Thank you for submitting your manuscript to PLOS Neglected Tropical Diseases. After careful consideration, we feel that it has merit but does not fully meet PLOS Neglected Tropical Diseases’s publication criteria as it currently stands. Therefore, we invite you to submit a revised version of the manuscript that addresses the points raised during the review process.

Please submit your revised manuscript within by Aug 15 2026 11:59PM. If you will need more time than this to complete your revisions, please reply to this message or contact the journal office at plosntds@plos.org. When you’re ready to submit your revision, log on to https://www.editorialmanager.com/pntd/ and select the 'Submissions Needing Revision' folder to locate your manuscript file.

Please include the following items when submitting your revised manuscript:

* A letter that responds to each point raised by the editor and reviewer(s). You should upload this letter as a separate file labeled 'Response to Reviewers'. This file does not need to include responses to any formatting updates and technical items listed in the 'Journal Requirements' section below.

* A marked-up copy of your manuscript that highlights changes made to the original version. You should upload this as a separate file labeled 'Revised Manuscript with Track Changes'.

* An unmarked version of your revised paper without tracked changes. You should upload this as a separate file labeled 'Manuscript'.

If you would like to make changes to your financial disclosure, competing interests statement, or data availability statement, please make these updates within the submission form at the time of resubmission. Guidelines for resubmitting your figure files are available below the reviewer comments at the end of this letter.

As the corresponding author, your ORCID iD is verified in the submission system and will appear in the published article. PLOS supports the use of ORCID, and we encourage all coauthors to register for an ORCID iD and use it as well. Please encourage your coauthors to verify their ORCID iD within the submission system before final acceptance, as unverified ORCID iDs will not appear in the published article. Only the individual author can complete the verification step; PLOS staff cannot verify ORCID iDs on behalf of authors.

We look forward to receiving your revised manuscript.

Kind regards,

Ran Wang, M.D.

Academic Editor

PLOS Neglected Tropical Diseases

Abdallah Samy

Section Editor

PLOS Neglected Tropical Diseases

Shaden Kamhawi

co-Editor-in-Chief

PLOS Neglected Tropical Diseases

orcid.org/0000-0003-4304-636XX

Paul Brindley

co-Editor-in-Chief

PLOS Neglected Tropical Diseases

orcid.org/0000-0003-1765-0002

**Additional Editor Comments (if provided):**

Please read carefully all review comments and address all of them before reconsidering a revised version of your manuscript.

**Journal Requirements:**

If the reviewer comments include a recommendation to cite specific previously published works, please review and evaluate these publications to determine whether they are relevant and should be cited. There is no requirement to cite these works unless the editor has indicated otherwise.

1) Please ensure that the CRediT author contributions listed for every co-author are completed accurately and in full.

At this stage, the following Authors/Authors require contributions: Godfrey Nattoh, Philip M Armstrong, and Doug E Brackney. Please ensure that the full contributions of each author are acknowledged in the "Add/Edit/Remove Authors" section of our submission form.

The list of CRediT author contributions may be found here: https://journals.plos.org/plosntds/s/authorship#loc-author-contributions

2) Please provide an Author Summary. This should appear in your manuscript between the Abstract (if applicable) and the Introduction, and should be 150-200 words long. The aim should be to make your findings accessible to a wide audience that includes both scientists and non-scientists. Sample summaries can be found on our website under Submission Guidelines:

https://journals.plos.org/plosntds/s/submission-guidelines#loc-parts-of-a-submission

3) We do not publish any copyright or trademark symbols that usually accompany proprietary names, eg ©,  ®, or TM  (e.g. next to drug or reagent names). Therefore please remove all instances of trademark/copyright symbols throughout the text, including:

- TM on pages: 16, and 17.

4) In the online submission form, you indicated that All images were collected and stored by the corresponding author and can be accessed upon request.. All PLOS journals now require all data underlying the findings described in their manuscript to be freely available to other researchers, either

1. In a public repository

2. Within the manuscript itself

3. Uploaded as supplementary information.

This policy applies to all data except where public deposition would breach compliance with the protocol approved by your research ethics board. If your data cannot be made publicly available for ethical or legal reasons (e.g., public availability would compromise patient privacy), please explain your reasons by return email and your exemption request will be escalated to the editor for approval. Your exemption request will be handled independently and will not hold up the peer review process, but will need to be resolved should your manuscript be accepted for publication. One of the Editorial team will then be in touch if there are any issues.

5) Please amend your detailed Financial Disclosure statement. This is published with the article. It must therefore be completed in full sentences and contain the exact wording you wish to be published.

i) State the initials, alongside each funding source, of each author to receive each grant. For example: "This work was supported by the National Institutes of Health (####### to AM; ###### to CJ) and the National Science Foundation (###### to AM).".

If you did not receive any funding for this study, please simply state: u201cThe authors received no specific funding for this work.u201d

6) We have amended your Competing Interest statement to comply with journal style. We kindly ask that you double check the statement and let us know if anything is incorrect.

**Reviewers' Comments:**

Reviewer's Responses to Questions

**Key Review Criteria Required for Acceptance?**

As you describe the new analyses required for acceptance, please consider the following:

**Methods**

-Are the objectives of the study clearly articulated with a clear testable hypothesis stated?

-Is the study design appropriate to address the stated objectives?

-Is the population clearly described and appropriate for the hypothesis being tested?

-Is the sample size sufficient to ensure adequate power to address the hypothesis being tested?

-Were correct statistical analysis used to support conclusions?

-Are there concerns about ethical or regulatory requirements being met?

Reviewer #1: I may be wrong but I am not fully aware how this method will help in the control of dengue.

Reviewer #2: Methods are good

Reviewer #3: Objectives & Hypotheses: The objectives are clearly articulated. The implicit hypothesis that signal-amplification technologies can reveal early, hidden viral dynamics and population bottlenecks is systematically tested. Study Design: The comparative framework evaluating HCR-IF, HCR-FISH, and HCR-FlowFISH directly against traditional IFA is exceptionally robust and logically sound. Sample Population: The choice of Aedes aegypti (Orlando strain) and DENV-2 (VENE93 strain) is standard and appropriate for vector competence evaluations. Statistical Power & Analysis: Non-parametric statistical approaches (Kruskal-Wallis with post-hoc multiple comparisons and Fisher’s exact test) are well-selected and applied properly given the distribution patterns of viral infection foci.

Ethical Requirements: The research utilizes commercially sourced defibrinated sheep's blood via artificial membrane feeders for mosquito colony maintenance and experimental infections. There are no human subjects or live vertebrate animal ethical concerns.

Methodological Clarification Required: Under the "Hybridization Chain Reaction-Flow Cytometry-FISH" section, the authors state that midguts were grouped in pools of five. Please explicitly declare how many independent pools (biological replicates) were run per timepoint to generate the scatter graphs and statistical estimates.

**********

**Results**

-Does the analysis presented match the analysis plan?

-Are the results clearly and completely presented?

-Are the figures (Tables, Images) of sufficient quality for clarity?

Reviewer #1: It is a new method to determine the dengue virus at a very early stage. I have nothing against it. But Ae. aegypti is a known as a good dengue vector. How is this method going to be useful in the future. perhaps this needs to be explained

Reviewer #2: results are good. Also I might have missed it... In the title it says "population bottlenecks AND temporal constraints shape dengue virus infection... what exactly is the population bottleneck you speak of? Is that in relation to the low number of cells infected in the midgut via your more precise method? I'm not exactly sure how you can jump to a conclusion about population bottlenecks with a single strain of Aedes aegypti maintained in a lab colony since 1952 - that colony IS a bottleneck.

Reviewer #3: Alignment with Plan: The presented results align cleanly with the stated comparative and kinetic objectives.

Clarity of Presentation: The results are highly readable, well-structured, and explicitly present both focus-forming counts and single-cell percentages.

Figure Quality: The schematic panels generated via BioRender clarify experimental workflows beautifully. Confocal micrographs present sharp spatial resolution, and the inset windows are exceptionally helpful for visualizing low-abundance signals.

**********

**Conclusions**

-Are the conclusions supported by the data presented?

-Are the limitations of analysis clearly described?

-Do the authors discuss how these data can be helpful to advance our understanding of the topic under study?

-Is public health relevance addressed?

Reviewer #1: This is something that i am not convinced. I may be wrong but I feel there should be more explanation on why this study was carried out.

Reviewer #2: conclusions are good. However, I am curious, are there any concerns regarding this novel technique in relation to cross reactivity? Can you be certain it's a dengue virus and not something else, like an enteric viral infection found in the colony? This can occasionally even happen with PCR and ELISA assays - cross reactivity.

Reviewer #3: Data Support: The conclusions regarding a severe early midgut bottleneck and asynchronous viral entry window are strongly supported by the statistical divergence between HCR tools and IFA.

Limitations Addressed: The authors properly acknowledge the challenges of distinguishing initial multi-virion clusters from expanding lateral foci at ultra-early intervals (6–12 hpi).

Public Health & Field Advancement: The manuscript emphasizes that identifying early cellular interactions can assist in developing targeted transmission-blocking strategies, establishing solid translational relevance.

**********

**Editorial and Data Presentation Modifications?**

Use this section for editorial suggestions as well as relatively minor modifications of existing data that would enhance clarity. If the only modifications needed are minor and/or editorial, you may wish to recommend “Minor Revision” or “Accept”.

Reviewer #1: nil

Reviewer #2: (No Response)

Reviewer #3: 1. Data Availability Statement: In the metadata table, the authors state: "All images were collected and stored by the corresponding author and can be accessed upon request."

Correction: This is non-compliant with PLOS Data Policies. Authors are required to make all minimal underlying data fully available without restriction. The raw numerical values behind graphs (Figures 1, 3, 4, and 5) must be uploaded as Supporting Information spreadsheets or deposited into a public repository (e.g., Dryad, Zenodo, or Figshare), and a repository accession link/DOI must be provided prior to final acceptance.

2. Terminology Consistency: On pages 19 and 20, the manuscript alternates somewhat inconsistently between using "hpi" (hours post-infection) and "hpbf" (hours post-blood feeding). Correction: Ensure standard, unified use of terminology throughout the text. If the authors view "hpi" and "hpbf" as interchangeable in this experimental layout, a single defining sentence should declare this early in the text to avoid reader confusion.

**********

**Summary and General Comments**

Use this section to provide overall comments, discuss strengths/weaknesses of the study, novelty, significance, general execution and scholarship. You may also include additional comments for the author, including concerns about dual publication, research ethics, or publication ethics. If requesting major revision, please articulate the new experiments that are needed.

Reviewer #1: It is a very interesting study and details have been provided. However, I have a few questions.

Ae. aegypti is a well know vector for dengue virus. Here you have used a special method to detect the virus at 6hr after ingestion of blood.

How will this method be used stop dengue transmission? This needs to be mentioned in more details.

Yes some scientists use RT-PCR to detect dengue virus in wild caught mosquitoes and they have reported that it could be a method for dengue control. However, we know that the control programme may find it difficult to carry out RT-PCR.

Now it has been established that that the NS1 antigen test kit can detect dengue virus in mosquitoes and this is a simple method that can be used by the control programme. It has also been established that there is a correlation between infected mosquitoes and DF. Besides once an infected mosquito is found dengue will occur lag of 1 to 2 weeks. Thus, action can be taken before cases are reported.

Thus, can you please explain how the method that you all have developed can be used to control an outbreak.

Ae. aegypti mosquitoes will lay eggs 48 to 72 hours after a blood meal. It is perhaps during this time that the mosquitoes can be easily captured.

I have nothing against the methods that you all have described. I am only interested in knowing how the transmission blocking strategy can be used?

Reviewer #2: I only had two concerns... the rest of the paper was well written. The discussion section and the pros in the use of this technique to elucidate viral infection time in support of vector competence studies was clever. I too am curious now why only a couple cells appear to become infected in the midgut when there are loads of virus and apparently plenty of cells to infect. Not sure about the hypothesis that viruses infect the cells that are concentrated in the caudal region to allow water to be filtered though... then that would propose mosquitoes that do not feed to repletion and therefore do not concentrate cells/virus in the caudal region of the posterior midgut have a lower propensity of infection... food for thought.

Reviewer #3: Overall Evaluation:

This manuscript addresses a significant and long-standing limitation in the field of arbovirology: the inability of traditional assays to detect and quantify initial viral infection events in mosquito vectors due to sensitivity constraints. By implementing hybridization chain reaction (HCR) coupled with immunofluorescence (HCR-IF), fluorescence in situ hybridization (HCR-FISH), and flow cytometry (HCR-FlowFISH), the authors successfully track dengue virus serotype 2 (DENV-2) kinetics in Aedes aegypti midguts at ultra-early timepoints (6, 12, 18, and 24 hours post-infection). The study provides clear empirical evidence of severe population bottlenecks, showing that only a minute fraction of ingested virions successfully initiate midgut epithelial infection. Furthermore, it identifies a strong spatial preference for infection initialization in the caudal section of the posterior midgut.

Strengths:

Methodological Innovation: The application of HCR signal amplification to the mosquito midgut provides an outstanding, highly sensitive alternative to classical immunofluorescence assays (IFA) and destructive PCR methods. Biological Insight: Capturing active replication events as early as 6 to 12 hours post-infection shifts our understanding of early arboviral intra-vector dynamics.

Experimental Rigor: The combination of confocal imaging quantification alongside single-cell dissociation and flow cytometry validation (HCR-FlowFISH) offers excellent data triangulation.

Weaknesses:

Data Availability Policy Violation: The current data availability statement ("accessible upon request") directly violates PLOS repository guidelines.

Flow Cytometry Sample Clarification: The replication layout and exact biological sample configuration for the flow cytometry pools require explicit clarification in the text.

**********

PLOS authors have the option to publish the peer review history of their article (what does this mean?). If published, this will include your full peer review and any attached files.

If you choose “no”, your identity will remain anonymous but your review may still be made public.

**Do you want your identity to be public for this peer review?**  For information about this choice, including consent withdrawal, please see our Privacy Policy.

Reviewer #1: No

Reviewer #2: No

Reviewer #3: No

[NOTE: If reviewer comments were submitted as an attachment file, they will be attached to this email and accessible via the submission site. Please log into your account, locate the manuscript record, and check for the action link "View Attachments". If this link does not appear, there are no attachment files.]

**Figure resubmission:** -->While revising your submission, we strongly recommend that you use PLOS’s NAAS tool (https://ngplosjournals.pagemajik.ai/artanalysis) to test your figure files. NAAS can convert your figure files to the TIFF file type and meet basic requirements (such as print size, resolution), or provide you with a report on issues that do not meet our requirements and that NAAS cannot fix.

After uploading your figures to PLOS’s NAAS tool - https://ngplosjournals.pagemajik.ai/artanalysis, NAAS will process the files provided and display the results in the "Uploaded Files" section of the page as the processing is complete. If the uploaded figures meet our requirements (or NAAS is able to fix the files to meet our requirements), the figure will be marked as "fixed" above. If NAAS is unable to fix the files, a red "failed" label will appear above. When NAAS has confirmed that the figure files meet our requirements, please download the file via the download option, and include these NAAS processed figure files when submitting your revised manuscript.

**Reproducibility:**

To enhance the reproducibility of your results, we recommend that authors of applicable studies deposit laboratory protocols in protocols.io, where a protocol can be assigned its own identifier (DOI) such that it can be cited independently in the future. Additionally, PLOS ONE offers an option to publish peer-reviewed clinical study protocols. Read more information on sharing protocols at https://plos.org/protocols?utm_medium=editorial-email&utm_source=authorletters&utm_campaign=protocols

# Author comment: Author response to Decision Letter 1

**DOI:** 10.1371/journal.pntd.0014592.r002

26 Jun 2026

Attachment Submitted filename: Response to reviewers.docx

# Editor report: Decision Letter 1

**DOI:** 10.1371/journal.pntd.0014592.r003

**Authors:** Abdallah Samy, Ran Wang

20 Jul 2026

Dear Dr Brackney,

We are pleased to inform you that your manuscript 'Population bottlenecks and temporal constraints shape dengue virus infection of Aedes aegypti midguts' has been provisionally accepted for publication in PLOS Neglected Tropical Diseases.

Before your manuscript can be formally accepted you will need to complete some formatting changes, which you will receive in a follow up email. A member of our team will be in touch with a set of requests.

Please note that your manuscript will not be scheduled for publication until you have made the required changes, so a swift response is appreciated.

IMPORTANT: The editorial review process is now complete. PLOS will only permit corrections to spelling, formatting or significant scientific errors from this point onwards. Requests for major changes, or any which affect the scientific understanding of your work, will cause delays to the publication date of your manuscript.

Should you, your institution's press office or the journal office choose to press release your paper, you will automatically be opted out of early publication. We ask that you notify us now if you or your institution is planning to press release the article. All press must be co-ordinated with PLOS.

Thank you again for supporting Open Access publishing; we are looking forward to publishing your work in PLOS Neglected Tropical Diseases.

Best regards,

Ran Wang, M.D.

Academic Editor

PLOS Neglected Tropical Diseases

Abdallah Samy

Section Editor

PLOS Neglected Tropical Diseases

Shaden Kamhawi

co-Editor-in-Chief

PLOS Neglected Tropical Diseases

orcid.org/0000-0003-4304-636XX

Paul Brindley

co-Editor-in-Chief

PLOS Neglected Tropical Diseases

orcid.org/0000-0003-1765-0002

***********************************************************

p.p1 {margin: 0.0px 0.0px 0.0px 0.0px; line-height: 16.0px; font: 14.0px Arial; color: #323333; -webkit-text-stroke: #323333}span.s1 {font-kerning: none

# Editor report: Acceptance letter

**DOI:** 10.1371/journal.pntd.0014592.r004

**Authors:** Abdallah Samy, Ran Wang

Dear Dr Brackney,

We are delighted to inform you that your manuscript, "Population bottlenecks and temporal constraints shape dengue virus infection of Aedes aegypti midguts," has been formally accepted for publication in PLOS Neglected Tropical Diseases.

We have now passed your article onto the PLOS Production Department who will complete the rest of the publication process. All authors will receive a confirmation email upon publication.

The corresponding author will soon be receiving a typeset proof for review, to ensure errors have not been introduced during production. Please review the PDF proof of your manuscript carefully, as this is the last chance to correct any scientific or type-setting errors. Please note that major changes, or those which affect the scientific understanding of the work, will likely cause delays to the publication date of your manuscript. Note: Proofs for Front Matter articles (Editorial, Viewpoint, Symposium, Review, etc...) are generated on a different schedule and may not be made available as quickly.

Soon after your final files are uploaded, the early version of your manuscript will be published online unless you opted out of this process. The date of the early version will be your article's publication date. The final article will be published to the same URL, and all versions of the paper will be accessible to readers.

For Research Articles, you will receive an invoice from PLOS for your publication fee after your manuscript has reached the completed accept phase. If you receive an email requesting payment before acceptance or for any other service, this may be a phishing scheme. Learn how to identify phishing emails and protect your accounts at https://explore.plos.org/phishing.

Thank you again for supporting open-access publishing; we are looking forward to publishing your work in PLOS Neglected Tropical Diseases.

Best regards,

Shaden Kamhawi

co-Editor-in-Chief

PLOS Neglected Tropical Diseases

Paul Brindley

co-Editor-in-Chief

PLOS Neglected Tropical Diseases
